# Patterns of medication use following breast cancer diagnosis: an Australian population-based study

**DOI:** 10.1007/s00520-025-09732-y

**Published:** 2025-07-08

**Authors:** Huah Shin Ng, Christoffer Johansen, Ming Li, David Roder, Kerri Beckmann, Bogda Koczwara

**Affiliations:** 1 College of Medicine and Public Health, Flinders Health and Medical Research Institute, Flinders University, Adelaide, South Australia Australia; 2 Cancer Epidemiology and Population Health Research Group, Allied Health and Human Performance, University of South Australia, Adelaide, South Australia Australia; 3 SA Pharmacy, SA Health, Adelaide, South Australia Australia; 4 Center for Cancer Late Effect Research CASTLE, Department of Oncology, Rigshospitalet, University of Copenhagen, Copenhagen, Denmark; 5 Faculty of Medicine, University of Queensland, Brisbane, QLD Australia; 6 Department of Medical Oncology, Flinders Medical Centre, Adelaide, South Australia Australia

**Keywords:** Breast cancer, Polypharmacy, Medication use, Population-based, South Australia

## Abstract

**Purpose:**

This study aimed to examine patterns of medication use and polypharmacy following breast cancer diagnosis.

**Methods:**

This retrospective cohort study used breast cancer data from the South Australian Cancer Registry linked with medication dispensing records, death registry, and inpatient hospital records. Women diagnosed with invasive breast cancer between July 2012 and March 2014 were followed for 5 years from diagnosis. All medications were defined using the Anatomical Therapeutic Chemical classification, and patterns of use were analysed in one-yearly intervals. The changes in the use of medications and polypharmacy (≥ 5 concomitant medications versus not) from Year-2 to Year-5 of breast cancer diagnosis were examined using generalised estimating equations models with binary logistic distribution.

**Results:**

The study included 2005 women (mean age = 61.1 years). The use of endocrine therapy for breast cancer decreased over time (odds ratio (OR) 0.88; 95%CI = 0.86–0.90). In contrast, the likelihood of being dispensed specific cardiovascular medicines increased with each successive time period including agents acting on renin-angiotensin system (OR 1.03; 95%CI = 1.01–1.05), lipid-modifying agents (OR 1.06; 95%CI = 1.03–1.08), beta-blockers (OR 1.08; 95%CI = 1.04–1.11), and cardiac therapy (OR 1.12; 95%CI = 1.06–1.18). There was an increased likelihood of polypharmacy over time (OR 1.08; 95%CI = 1.04–1.11) with the prevalence ranging from 25% (Year 2) to 29% (Year 5). Several characteristics were associated with polypharmacy including older age, a lower socioeconomic status, and a higher burden of comorbidities.

**Conclusion:**

The use of several medication classes increased over time suggesting development of new comorbidities and higher likelihood of polypharmacy. Medication management in breast cancer survivors offers potential to identify those with complex needs of polypharmacy and comorbidity.

**Supplementary Information:**

The online version contains supplementary material available at 10.1007/s00520-025-09732-y.

## Introduction

Breast cancer is the most common cancer diagnosed in women in Australia, with excellent 5-year relative survival of 92% (2014–2018) [1]. Individuals with breast cancer are also at risk of dying from other health conditions, particularly cardiovascular diseases which are the leading cause of non-cancer death among survivors [2, 3]. As many women with breast cancer survive long-term [4], the management of these other chronic diseases (‘comorbidities’) is recognised as an important part of the survivorship care [5]. Our previous study, which used medication dispensing records as proxy measures for comorbidities, showed that Australian women with hormone-dependent breast cancer were more likely to develop a range of health conditions including depression, pain/pain-inflammation, osteoporosis, diabetes, cardiovascular conditions, and gastric disorders compared with the general population without cancer [6]. However, our survey of Australian breast cancer survivors showed that the management of comorbidities was inadequately aligned with the established chronic disease management principles [7].

Cancer survivors often take multiple medications for their comorbidities in addition to oral anti-cancer medication such as endocrine therapy for hormone-receptor positive breast cancer, so medication management is an important aspect of comorbidity management [6, 8]. Polypharmacy is described as the use of multiple medications by a person, and a cut-off of five or more medicines is often used as the numeric threshold to define polypharmacy [9]. Polypharmacy has been associated with inferior health outcomes including increased mortality, hospitalisations, and adverse drug reactions in cancer patients [10–12]. Previous studies on the impact of polypharmacy on medication adherence in cancer patients showed mixed findings, with some studies demonstrated that polypharmacy was associated with a greater medication adherence [13, 14], while others found the opposite [15]. Prior studies have examined the prevalence of polypharmacy in patients with breast cancer; however, they were limited by the measurement of polypharmacy at a single time point [8, 16]. Little is known about the changes in the patterns of medication use and polypharmacy within the first few years of breast cancer diagnosis.

The aim of this study was to examine the patterns of medication use in women with breast cancer within 5 years from the date of diagnosis, and the characteristics associated with polypharmacy.

## Methods

### Data source and study population

We conducted this retrospective cohort study using the breast cancer data from the South Australian Cancer Registry linked with administrative health data from medication dispensing records, the National Death Index (NDI), and inpatient hospital records. Reporting of all cancer cases diagnosed in the State including breast cancers to the South Australian Cancer Registry is mandated by law. The data obtained from the South Australian Cancer Registry included age at breast cancer diagnosis, demographics (e.g., country of birth, geographical location, socioeconomic status), histology (e.g., ductal, lobular), and differentiation (e.g., low, intermediate, high) of breast cancer. The medication dispensing records from the Pharmaceutical Benefits Scheme (PBS) captured prescription medications subsidised by the Australian Government to eligible citizens and residents and included information such as date of supply, medication name, strength, and Anatomical Therapeutic Chemical (ATC) classification. Over-the-counter medications, vitamins, complementary medicines, medications given as inpatients at public hospitals, and medications written as ‘private’ prescriptions (e.g., medications not listed under PBS schedule) were not captured in the PBS data. Prior to July 2012, the PBS data also did not include dispensing for medications that fell below the consumer copayment level. Therefore, we used PBS dispensing records from July 2012 onwards to ensure more complete capture of medication use within our study population and we had access to the dispensing records up to March 2019 for this study. The NDI captured date of death, and inpatient hospital data provided information on hospital admissions/discharges with diagnoses coded using the International Classification Diseases (ICD) codes.

We identified all women diagnosed with invasive breast cancer between July 2012 and March 2014 who had survived for 5 years from breast cancer diagnosis as we were interested in long-term survivors and acknowledged that use of medications for those with shorter life expectancy may vary due to differences in goal of care [17]. The study population were followed for 5 years from breast cancer diagnosis.

### Outcome measures

(i) Specific medication use
  We identified all medications dispensed within 5 years from the date of breast cancer diagnosis using the ATC classification system by therapeutic or pharmacological subgroups (i.e., ATC second level) in the PBS data. The patterns of medication use by ATC second level (‘medication class’) were analysed in one-yearly time intervals. Prevalence of specific medication use was calculated by dividing the number of patients who were dispensed a particular medication class at least once during each 1-year time interval by the total number of study population. We reported specific medication classes with a prevalence of 5% or more during each 1-year time period in our study population because of statistical power considerations.
(ii) Polypharmacy
  We calculated polypharmacy based on the number of unique medications dispensed in one-yearly time intervals. Polypharmacy was defined as having five or more unique medications identified using ATC classification fifth level (by chemical substance) in the PBS data. As we are interested in chronic medication use, a medication had to be dispensed at least four times in each 1-year time interval to be included in the count of polypharmacy. Endocrine therapy for breast cancer was considered in the count of polypharmacy, but other antineoplastic agents (ATC L01 e.g., chemotherapy, monoclonal antibodies) were not. Prevalence of polypharmacy was calculated by dividing the number of patients who were dispensed five or more unique medications during each 1-year time interval by the total number of study population.

### Other variables

Data on the patient characteristics at breast cancer diagnosis were extracted: age (categorised as < 50, 50–59, 60–69, 70–79, or ≥ 80 years), country of birth (categorised as Australia, other mainly English-speaking countries, mainly non-English-speaking countries or unknown), geographical location (identified using the Australian Standard Geographical Classification Remoteness Index and categorised as major cities, inner regional, or outer and remote areas) [18], socioeconomic status (derived from the residential postcode using the Socioeconomic Index for Areas (SEIFA) Index of Relative Socioeconomic Disadvantage and expressed as quintiles) [19], histology (categorised as ductal, lobular, other or unknown), and differentiation of breast cancer (categorised as low, intermediate, high or unknown). The burden of comorbidity at breast cancer diagnosis was measured by the Charlson Comorbidity Index (CCI) [20], which was derived from inpatient hospital data using records up to 5 years prior to breast cancer diagnosis, and categorised as 0, 1, or ≥ 2.

### Statistical analysis

Characteristics of the study population and the prevalence of medication use were described using descriptive analysis including frequency and percentage for categorical variables and mean and standard deviation, or median, first quartile (Q1) and third quartile (Q3) for continuous variables as appropriate.

We described the prevalence of medication use over 5 years of breast cancer diagnosis. The medication use in the first year of breast cancer diagnosis comprised a range of supportive care treatments that differed from the patterns of medication uses for subsequent years. Therefore, we assessed post-diagnosis changes in prevalence of medication use by medication classes (with a dispensing record versus no record for each year) and polypharmacy (using ≥ 5 concomitant medications versus not) over time from Year 2 to Year 5 following breast cancer diagnosis using the generalised estimating equations (GEE) models with binary logistic distribution. The GEE used a robust variance estimator and unstructured correlation matrix with binomial distribution and logit link. Time period (i.e., four one-yearly time intervals) was included as continuous variable. The results were reported as odds ratios (ORs) with 95% confidence intervals (CIs).

To assess the characteristics associated with polypharmacy, a multivariable GEE model was also used considering the following factors: age at breast cancer diagnosis, country of birth, geographical location, socioeconomic status, histology, differentiation of breast cancer, comorbidity burden, and time period (as continuous variable comprising four one-yearly time intervals from Year 2 to Year 5 post-breast cancer diagnosis). We conducted sensitivity analyses by including medications that were dispensed at least two times or three times (as opposed to at least four times in the primary analysis) in each 1-year time interval in the count of polypharmacy (i.e., having five or more unique medications with each medication dispensed two or three times).

We also explored the characteristics associated with the likelihood of being dispensed an endocrine therapy for breast cancer using the multivariable GEE model considering the same factors listed above and by excluding endocrine therapy in the count of polypharmacy (i.e., having five or more unique medications (excluding endocrine therapy) with each medication dispensed at least four times). We also described the prevalence of polypharmacy and number of medicine use based on dispensing records of 1 year prior to breast cancer diagnosis, in women exposed and unexposed to endocrine therapy in the first year of breast cancer diagnosis.

We used a latent class analysis to explore the patterns of medication use by ATC second level in Year 5 and trialled models containing between two and six classes. A three-class model was selected based on Bayesian information criteria (BIC) statistics and model parsimony [21]. We then subjectively labelled each class according to the most discriminating medication types.

All statistical analyses were performed using the SAS software version 9.4 (Cary, NC, USA).

## Ethics approval

The study was approved by the ethics committee of the SA Heath (2020/HRE00389), the University of South Australia (#200,021), and the Australian Institute of Health and Welfare (EO2017/3/361). The study was performed in accordance with the ethical standards of the 1964 Declaration of Helsinki and its later amendments.

## Results

### Cohort characteristics

We identified 2338 women diagnosed with invasive breast cancer between July 2012 and March 2014, with 333 of these women dying within 5 years of breast cancer diagnosis. A total of 2005 women (mean age = 61.1 years; SD = 12.8 years) who survived for 5 years from breast cancer diagnosis were included in the final analysis. The majority of the study population were born in Australia (66%), resided in major cities (74%), and had a low burden of comorbidity (9% with comorbidity measured by the CCI) at the time of diagnosis (Table 1).

**Table 1:** Characteristics of the study population, *N* = 2005

Characteristics	*n* (%)
Age at diagnosis in years	
< 50 50–59 60–69 70–79 ≥ 80	396 (20) 506 (25) 580 (29) 337 (17) 186 (9)
Country of birth	
Australia Other mainly English-speaking countries Mainly non-English-speaking countries Unknown	1329 (66) 311 (16) 259 (13) 106 (5)
Geographical location	
Major cities Inner regional Outer and remote	1487 (74) 212 (11) 306 (15)
Socioeconomic status	
1 (lowest) 2 3 4 5 (highest)	370 (18) 429 (21) 355 (18) 427 (21) 424 (21)
Histology	
Ductal Lobular Other Unknown	1506 (75) 210 (11) 270 (13) 19 (1)
Differentiation	
Low Intermediate High Unknown	651 (32) 910 (45) 349 (17) 95 (5)
Comorbidity burden (Charlson comorbidity index, excluding breast cancer in the count)	
0 1 ≥ 2	1828 (91) 91 (5) 86 (4)

### Prevalence of medication use

A total of 373,536 prescriptions were dispensed to the study population over the 5-year follow-up period, with a median of 148 (Q1–Q3 76–265) prescriptions over 5 years and a median of 18 (Q1–Q3 12–25) unique medications (total *n* = 37,941 medicines) per patient. Systemic antibacterial agents were the most prevalent medication class dispensed at least once for 91% (*n* = 1830/2005) of the study population, followed by endocrine therapy for 76% (*n* = 1516), analgesics for 74% (*n* = 1483), and drugs for acid-related disorders for 57% (*n* = 1133) of the study population during the 5-year follow-up period.

About one-third (31%; *n* = 11,894/37,941) of medicines were dispensed to the study population once only during the follow-up period, 13% (*n* = 5085) were dispensed twice, 22% (*n* = 8228) three to five times, and 34% (*n* = 12,734) six times or more.

The most prevalent medication classes dispensed during the first year of diagnosis were endocrine therapy for breast cancer (73%), followed by systemic antibacterial agents (66%), analgesics (53%), antiemetics/antinauseants (45%), and drugs for acid-related disorders (42%) (Table S1). The prevalence of medication use over each of the 5-year period was shown in Table S1.

### Changes in medication use over time (Year 2–Year 5)

The use of endocrine therapy for breast cancer (OR 0.88; 95%CI = 0.86–0.90) and analgesics (OR 0.88; 95%CI = 0.85–0.91) decreased by 12% over time (Table 2). The likelihood of being dispensed selected cardiovascular medicines increased with each successive time period (from Year 2 to Year 5 following breast cancer diagnosis) and varied by types of medication classes ranging from 3% for agents acting on renin-angiotensin system (OR 1.03; 95%CI = 1.01–1.05), 6% for lipid-modifying agents (OR 1.06; 95%CI = 1.03–1.08), 8% for beta-blockers (OR 1.08; 95%CI = 1.04–1.11), through to 12% for cardiac therapy (OR 1.12; 95%CI = 1.06–1.18). The medication uses for thyroid (OR 1.04; 95%CI = 1.02–1.07), diabetes (OR 1.08; 95%CI = 1.05–1.11), obstructive airway (OR 1.09; 95%CI = 1.05–1.13), and bone diseases (OR 1.12; 95%CI = 1.08–1.17) also increased over time. There were no significant changes in other medication uses including systemic antibacterial agents, psycholeptics (e.g., antipsychotics, anxiolytics, hypnotics, and sedatives), psychoanaleptics (e.g., antidepressants, and anti-dementia drugs), antirheumatic, and anti-anaemic preparations over time.

**Table 2:** Likelihood of medication use over time (Year 2–Year 5) in women with breast cancer post-diagnosis

Medication classes	ATC code	Odds ratio (95% CI)	*p*-value
Endocrine therapy	L02	0.88 (0.86–0.90)*	< 0.0001
Analgesics	N02	0.88 (0.85–0.91)*	< 0.0001
Antithrombotic agents	B01	0.98 (0.94–1.03)	0.4402
Ophthalmological	S01	0.98 (0.93–1.03)	0.4598
Anti-inflammatory and antirheumatic products	M01	0.99 (0.95–1.03)	0.5528
Antibacterial for systemic use	J01	1.00 (0.97–1.03)	0.9776
Psychoanaleptics	N06	1.00 (0.97–1.02)	0.8260
Corticosteroids, dermatological preparations	D07	1.00 (0.96–1.05)	0.9247
Psycholeptics	N05	1.01 (0.97–1.04)	0.7381
Sex hormones and modulators of the genital system	G03	1.01 (0.96–1.07)	0.6599
Anti-anaemic preparations	B03	1.02 (0.95–1.09)	0.5930
Antiemetics and antinauseants	A04	1.06 (0.99–1.13)	0.0998
Agents acting on the renin-angiotensin system	C09	1.03 (1.01–1.05)*	0.0008
Diuretics	C03	1.04 (1.00–1.08)*	0.0459
Thyroid therapy	H03	1.04 (1.02–1.07)*	0.0007
Calcium channel blockers	C08	1.04 (1.00–1.09)*	0.0392
Drugs for acid related disorders	A02	1.05 (1.02–1.08)*	0.0003
Lipid modifying agents	C10	1.06 (1.03–1.08)*	< 0.0001
Beta blocking agents	C07	1.08 (1.04–1.11)*	< 0.0001
Drugs used in diabetes	A10	1.08 (1.05–1.11)*	< 0.0001
Drugs for obstructive airway disease	R03	1.09 (1.05–1.13)*	< 0.0001
Corticosteroids for systemic use	H02	1.10 (1.04–1.16)*	0.0012
Drugs for functional gastrointestinal disorders	A03	1.10 (1.02–1.19)*	0.0193
Drugs for treatment of bone diseases	M05	1.12 (1.08–1.17)*	< 0.0001
Cardiac therapy	C01	1.12 (1.06–1.18)*	0.0001

Odds ratios are shown for the effect of time period on medication use (e.g., with each successive time period, the likelihood of being dispensed agents acting on the renin-angiotensin system increased by 3%). Time period (i.e., four one-yearly time intervals) was included as continuous variable from Year 2 to Year 5 post-breast cancer diagnosis

^*^p-value < 0.05

### Polypharmacy

The prevalence of polypharmacy ranged from 25% (Year 2) to 29% (Year 5). There was an increased likelihood in polypharmacy over time (aOR 1.08; 95%CI = 1.04–1.11) (Table 3). The direction and magnitude of the findings were similar using two (aOR 1.05; 95%CI = 1.02–1.08) or three times (aOR 1.07; 95%CI = 1.04–1.10) of dispensing for each medication during each 1-year interval in the count of polypharmacy. Several characteristics were associated with the likelihood of polypharmacy including older age, a lower socioeconomic status, and a higher burden of comorbidity.

**Table 3:** Characteristics associated with polypharmacy over time (Year 2-Year5)

Characteristics	Adjusted odds ratio	95% CI
Age at diagnosis in years		
< 50 50–59 60–69 70–79 ≥ 80	Reference 1.45 3.87 6.93 12.78	- 1.01–2.07* 2.79–5.38* 4.88–9.84* 8.52–19.17*
Country of birth		
Australia Other mainly English-speaking countries Mainly non-English-speaking countries Unknown	Reference 0.91 0.70 1.52	- 0.70–1.20 0.52–0.95* 1.03–2.25*
Geographical location		
Major cities Inner regional Outer and remote	Reference 1.14 1.05	- 0.82–1.58 0.80–1.39
Socioeconomic status		
1 (lowest) 2 3 4 5 (highest)	2.32 1.57 1.50 1.24 Reference	1.68–3.21* 1.14–2.17* 1.08–2.07* 0.91–1.70 -
Histology		
Ductal Lobular Other Unknown	Reference 0.80 1.07 0.23	- 0.58–1.11 0.80–1.42 0.09–0.64*
Differentiation		
Low Intermediate High Unknown	Reference 1.12 1.04 1.10	- 0.89–1.41 0.77–1.39 0.68–1.76
Comorbidity burden (Charlson comorbidity index, excluding breast cancer in the count)		
0 1 ≥ 2	Reference 3.01 3.34	- 1.95–4.63* 2.18–5.11*
Time period (yearly increment)	1.08	1.04–1.11*

^*^p-value < 0.05

Polypharmacy (by excluding endocrine therapy in the count of polypharmacy) (aOR 1.13; 95%CI = 1.04–1.22) was associated with an increased likelihood of being dispensed an endocrine therapy over time, while there were no significant differences in other factors including age, socioeconomic status, and comorbidity on endocrine therapy dispensing. The proportion of people with polypharmacy (determined using dispensing records in the year prior to breast cancer diagnosis) was higher among women treated with endocrine therapy in the first year (*n* = 267; 18%) compared to women unexposed to endocrine therapy (*n* = 56; 10%). The mean (SD) number of medicines dispensed at least four times in the year prior to breast cancer diagnosis was 2.4 (2.7) in women treated with endocrine therapy in the first year and 1.7 (2.5) in women unexposed to endocrine therapy.

### Patterns of medication use during Year 5

**Fig. 1:**
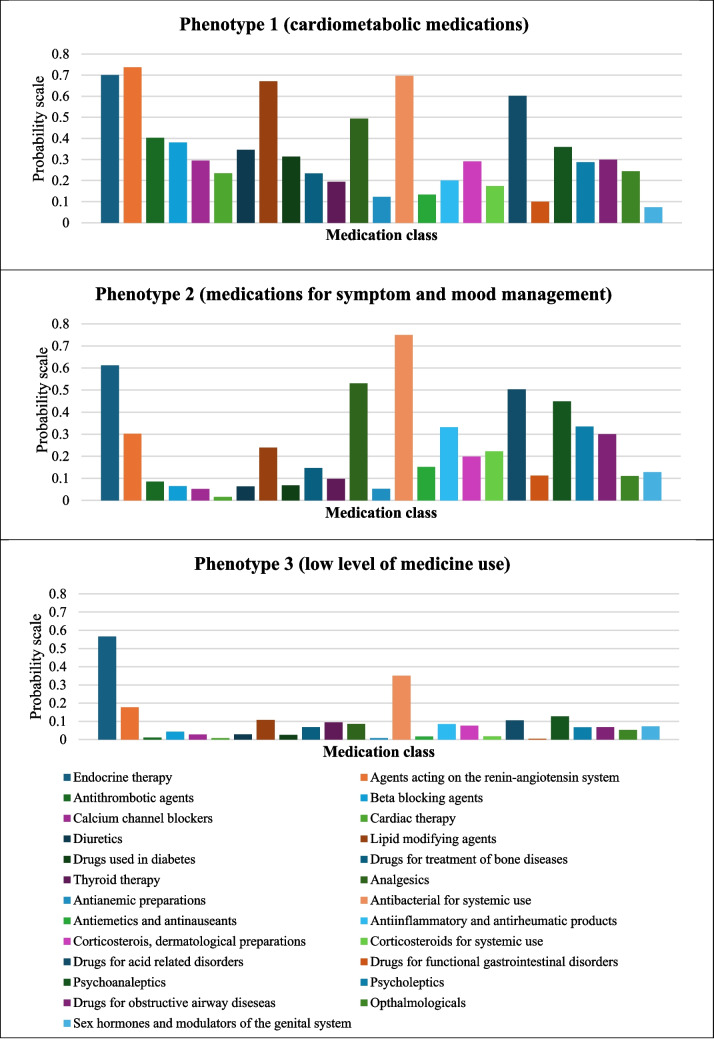
Patterns of medication use during year 5 of breast cancer diagnosis identified using latent class analysis

Three patterns of medication use during Year 5 were identified using latent class analysis and labelled as phenotype 1 (cardiometabolic medications; *n* = 524, 26%), phenotype 2 (medications for symptom and mood management such as analgesics and antidepressants; *n* = 555, 28%), and phenotype 3 (low level of medication use; *n* = 926, 46%) (Fig. 1). A higher proportion of people belonging to phenotype 1 were older (54% aged ≥ 70 years vs. 18% and 15%, in phenotypes 2 and 3, respectively) and had a higher burden of comorbidity measured by CCI (22% with CCI ≥ 1 vs. 7% and 3%) and a lower socioeconomic status (50% in quintile 1–2 vs. 41% and 33%) compared to phenotypes 2 and 3 (Table S2).

## Discussion

Three main findings arise from this population-based cohort study in women with breast cancer. First, while the use of endocrine therapy for breast cancer declined over time post-diagnosis, the use of specific cardiovascular medicines, and medications for metabolic disorders such as diabetes, thyroid, and bone diseases increased with each successive time period suggesting development of new co-morbid conditions. Second, there was an increased likelihood of polypharmacy over time (OR 1.08; 95%CI = 1.04–1.11) and the associated characteristics included an older age, a higher burden of comorbidity, and a lower socioeconomic status. Third, there were three patterns of medication use, with one-quarter of the study cohort having a high prevalence of cardiometabolic medications use, and one-quarter of people more likely to be using medications for symptom and mood management.

Despite the benefits of endocrine therapy in preventing recurrence of breast cancer or death [22], the likelihood of being dispensed an endocrine therapy in our study cohort decreased with each successive time period (OR 0.88; 95%CI = 0.86–0.90) consistent with literature elsewhere [23]. We found that polypharmacy (excluding endocrine therapy in the polypharmacy count) was associated with a higher likelihood of being dispensed an endocrine therapy over time suggesting that the drop in endocrine therapy use is unlikely to be driven by lack of access to health services, cost of drugs, or complexity of polypharmacy, but rather by other factors, for example endocrine therapy tolerance. This is to be expected in the Australian health care setting with universal access to health care and publicly funded subsidies for medications. Previous other studies from the USA and France also demonstrated that polypharmacy was associated with a greater adherence to endocrine therapy [13, 14, 24]. It is possible that the association between endocrine therapy adherence and polypharmacy is explained by greater engagement with the health system by those who receive multiple medications. In addition, endocrine therapy may contribute to polypharmacy with additional medications prescribed to manage side effects associated with endocrine therapy [13, 14, 24]. Further research exploring these issues in detail could help in designing interventions to improve endocrine therapy adherence.

Our study showed an increase in the dispensing of several types of cardiovascular and metabolic disease medications over time suggesting development of new comorbidities. This may be due to ageing, and shared risk factors between cancer and cardiovascular and other chronic diseases such as poor diet habit, physical inactivity, obesity, and smoking [25]. It is plausible that adverse effects from cancer treatment may lead to the development of new comorbidities, necessitating the need of additional medications to manage those conditions [6]. For example, treatment of breast cancer with chemotherapy (e.g., anthracycline), monoclonal antibodies (e.g., trastuzumab), and endocrine therapy is known to increase the risks of developing cardiovascular diseases [26, 27]. Endocrine therapy can also lead to osteopenia and osteoporosis, requiring treatment with pharmacological therapy such as bisphosphates [28]. This may partly explain our observations, which also showed a greater use of drugs for the treatment of bone diseases over time (OR 1.12; 95%CI = 1.08–1.17). An increased use of medications may also reflect a growing attention to diagnosing and addressing comorbidities due to more frequent contact with health care providers for management of breast cancer [29]. Our findings underscore the importance of comorbidity management in cancer survivors and the potential of medication management as a strategy to identify individuals with greatest/most complex needs. Our study identified a phenotype of people with a high prevalence of cardiometabolic medications representing a quarter of the study cohort, who were more likely to be older and have a higher burden of comorbidity and a lower socioeconomic status emphasising that these individuals at higher risk of cardiovascular disease. As cardiovascular disease is the leading cause of non-cancer death among breast cancer survivors [2, 3, 30, 31], our findings support the importance of cardiovascular disease risk assessment targeting high risk groups for early intervention [32–34].

We also identified a phenotype of people comprising over one-quarter of the study cohort with a high prevalence of medication use for symptom and mood management such as analgesics, antiemetics, drugs for acid related/gastrointestinal disorders, psycholeptics (e.g., anxiolytics, hypnotics, and sedatives), and psychoanaleptics (e.g., antidepressants). They were more likely to be younger and have a lower burden of comorbidity compared with the cardiometabolic phenotype and were evenly distributed across socioeconomic status. Increased physical symptoms such as pain or nausea and a younger age are the several individual risk factors identified to be associated with mood disorders including depression and anxiety in adult cancer patients [35]. Our findings support the need to manage comorbid symptoms that often coexist with anxiety or depression whereby the detection of one symptom should prompt the examination of the other condition [35]. The use of psychotropic drugs with other prescribed medications and potential clinically significant drug–drug interactions should also be considered as an important priority in this cohort [36].

The prevalence of polypharmacy in this study was lower than that of other studies in breast cancer although these prior studies were limited by small sample sizes and the measurement of polypharmacy at a single time point [8, 16], whereas our study included all women diagnosed with breast cancer in South Australia with their polypharmacy measured longitudinally over 5 years. The prevalence of 25–29% with polypharmacy observed in our study also differed and was generally higher from that of the general population in other western countries. For example, the polypharmacy prevalence in the general population ranged from 9.54–9.93% among New Zealanders [37] to 14.8–17.1% among US adults [38]. The 33% polypharmacy rates from general population of Danish adults were slightly higher than observed in our study [39]. Further studies are needed to compare the pattern and changes in medication use over time between cancer survivors and age-matched controls without cancer.

The likelihood of polypharmacy of our study cohort increased with each successive time period (aOR 1.08; 95%CI = 1.04–1.11), and factors associated with polypharmacy were older age, a higher burden of comorbidity and a lower socioeconomic status. The observed increase odds in polypharmacy by age and comorbidity burden are consistent with other studies in the general population [40, 41], and studies in cancer patients [42, 43]. Our finding was also in line with a recent systematic review, which showed socioeconomic inequalities in polypharmacy in the general elderly adults, with people of lower socioeconomic status having higher odds of polypharmacy [44]. Our findings suggest that factors such as socioeconomic status may be used as a prompt for medication management review services in cancer survivors. Assessment of medication use may serve as a good surrogate for symptom burden and comorbidity. It may be beneficial to adopt a multidisciplinary team approach of pharmacists working with other health professionals (e.g., oncologist, general practitioners, nurses, and allied health professionals) to manage polypharmacy and comorbidity, deprescribe, and simplify medication regimens for cancer survivors [45].

This study has several limitations. It is possible that we might have underestimated the prevalence of polypharmacy and the use of selected medications after breast cancer diagnosis, as over-the-counter medicines including analgesics and antithrombotic agents (e.g., aspirin used as antiplatelet for cardiovascular diseases) or private prescriptions were not captured in the dispensing records from the Pharmaceutical Benefits Scheme. We also do not have patient-specific clinical data such as functional and frailty status to assess if the individual medication regimens were therapeutic appropriate. While we were able to consider the burden of comorbidity using CCI, these comorbidity scores were relatively low in our study cohort. It would be of value for future studies to consider additional comorbidities not captured by the CCI but are common among breast cancer survivors such as lymphoedema, neuropathy, and osteoporosis [7]. Nonetheless, our findings provided the estimates on the prevalence and trend of multiple medication use and polypharmacy over a 5-year period in women with breast cancer by using prospectively collected population-based data, which was a major strength of the current study and was not subject to potential recall bias [46]. The use of dispensing/prescription medication data is a validated approach for estimating medication use [47]. Our study also demonstrated the value of data linkage, which enabled large-scale longitudinal data analyses of whole population across the state and the evaluation of medication use in the real-world setting [46] to inform potential improvements in delivery of care of cancer survivors.

## Conclusion

While endocrine therapy use decreased over time, the use of several medication classes in breast cancer survivors increased as did polypharmacy, which may indicate the development of new comorbidities with three distinct patterns of medication use—high cardiometabolic, high symptom management, and low medication use. Medication management in breast cancer survivors may offer potential to identify those with complex needs of polypharmacy and comorbidity.

## Supplementary Information

Below is the link to the electronic supplementary material.

Supplementary file 1 (DOCX 15.8 KB)

Supplementary file 2 (DOCX 15.4 KB)

## Data Availability

The data that support the findings of this study are available from the South Australian Department for Health and Wellbeing (South Australian Cancer Registry & Integrated South Australian Activity Collection) and the Australian Institute of Health and Welfare (PBS and NDI) but restrictions apply to the availability of these data, which were used under license for the current study, and so are not publicly available. Data are, however, available from the authors upon reasonable request and with permission of the South Australian Department for Health and Wellbeing and the Australian Institute of Health and Welfare.
