# Supplementary material for: Patterns of medication use following breast cancer diagnosis: an Australian population-based study
Source: Support Care Cancer. 2025 Jul 8;33(8):668. doi: 10.1007/s00520-025-09732-y (PMC12238185; doi:10.1007/s00520-025-09732-y)
Supplement: Supplementary file 1 — (DOCX 15.8 KB) [file 520_2025_9732_MOESM1_ESM.docx]

**Supplementary Table 1** The prevalence of commonly dispensed medication classes over time in women with breast cancer post-diagnosis

| **Medication classes** | **ATC code** | **Prevalence during Year 1 [months 1-12],**  **n (%)** | **Prevalence during Year 2 [months 13-24],**  **n (%)** | **Prevalence during Year 3 [months 25-36],**  **n (%)** | **Prevalence during Year 4 [months 37-48],**  **n (%)** | **Prevalence during Year 5 [months 49-60],**  **n (%)** |
| --- | --- | --- | --- | --- | --- | --- |
| Endocrine therapy | L02 | 1468 (73) | 1403 (70) | 1347 (67) | 1290 (64) | 1232 (61) |
| Analgesics | N02 | 1064 (53) | 820 (41) | 757 (38) | 674 (34) | 647 (32) |
| Antithrombotic agents | B01 | 328 (16) | 283 (14) | 277 (14) | 248 (12) | 275 (14) |
| Ophthalmological | S01 | 261 (13) | 257 (13) | 229 (11) | 214 (11) | 242 (12) |
| Anti-inflammatory and antirheumatic products | M01 | 392 (20) | 385 (19) | 387 (19) | 380 (19) | 374 (19) |
| Antibacterial for systemic use | J01 | 1315 (66) | 1123 (56) | 1084 (54) | 1095 (55) | 1118 (56) |
| Psychoanaleptics | N06 | 522 (26) | 567 (28) | 574 (29) | 577 (29) | 565 (28) |
| Corticosteroids, dermatological preparations | D07 | 482 (24) | 337 (17) | 294 (15) | 304 (15) | 338 (17) |
| Psycholeptics | N05 | 573 (29) | 400 (20) | 374 (19) | 370 (18) | 406 (20) |
| Sex hormones and modulators of the genital system | G03 | 227 (11) | 174 (9) | 167 (8) | 172 (9) | 178 (9) |
| Anti-anaemic preparations | B03 | 92 (5) | 98 (5) | 106 (5) | 105 (5) | 103 (5) |
| Antiemetics and antinauseants | A04 | 906 (45) | 145 (7) | 145 (7) | 136 (7) | 173 (9) |
| Agents acting on the renin-angiotensin system | C09 | 691 (34) | 681 (34) | 698 (35) | 712 (36) | 727 (36) |
| Diuretics | C03 | 249 (12) | 219 (11) | 236 (12) | 228 (11) | 248 (12) |
| Thyroid therapy | H03 | 213 (11) | 220 (11) | 229 (11) | 244 (12) | 245 (12) |
| Calcium channel blockers | C08 | 190 (9) | 190 (9) | 193 (10) | 210 (10) | 212 (11) |
| Drugs for acid related disorders | A02 | 848 (42) | 643 (32) | 662 (33) | 677 (34) | 707 (35) |
| Lipid modifying agents | C10 | 522 (26) | 527 (26) | 548 (27) | 580 (29) | 594 (30) |
| Beta blocking agents | C07 | 217 (11) | 230 (11) | 245 (12) | 258 (13) | 280 (14) |
| Drugs used in diabetes | A10 | 182 (9) | 186 (9) | 205 (10) | 216 (11) | 230 (11) |
| Drugs for obstructive airway disease | R03 | 331 (17) | 310 (15) | 377 (19) | 371 (19) | 394 (20) |
| Corticosteroids for systemic use | H02 | 621 (31) | 179 (9) | 201 (10) | 190 (9) | 237 (12) |
| Drugs for functional gastrointestinal disorders | A03 | 524 (26) | 96 (5) | 102 (5) | 119 (6) | 123 (6) |
| Drugs for treatment of bone diseases | M05 | 154 (8) | 200 (10) | 220 (11) | 242 (12) | 270 (13) |
| Cardiac therapy | C01 | 94 (5) | 102 (5) | 120 (6) | 124 (6) | 141 (7) |
