# Supplementary material for: Patterns of medication use following breast cancer diagnosis: an Australian population-based study
Source: Support Care Cancer. 2025 Jul 8;33(8):668. doi: 10.1007/s00520-025-09732-y (PMC12238185; doi:10.1007/s00520-025-09732-y)
Supplement: Supplementary file 2 — (DOCX 15.4 KB) [file 520_2025_9732_MOESM2_ESM.docx]

**Supplementary Table 2** Characteristics of the study population by phenotypes identified using latent class analysis

| **Characteristics** | **Phenotype 1**  **(n=524)** | **Phenotype 2**  **(n=555)** | **Phenotype 3**  **(n=926)** | **P-value** |
| --- | --- | --- | --- | --- |
|  | **Cardio-metabolic medications** | **Medications for symptom and mood management** | **Low level of medication use** |  |
|  | **n (%)** | | | |
| **Age at diagnosis in years**  <50  50-59  60-69  70-79  ≥80 | 16 (3)  57 (11)  167 (32)  174 (33)  110 (21) | 122 (22)  148 (27)  185 (33)  64 (12)  36 (6) | 258 (28)  301 (32)  228 (25)  99 (11)  40 (4) | <0.0001 |
| **Country of birth**  Australia  Other mainly English-speaking countries  Mainly non-English-speaking countries  Unknown | 328 (63)  95 (18)  69 (13)  32 (6) | 387 (70)  80 (14)  61 (11)  27 (5) | 614 (66)  136 (15)  129 (14)  47 (5) | 0.2031 |
| **Geographical location**  Major cities  Inner regional  Outer and remote | 373 (71)  56 (11)  95 (18) | 411 (74)  59 (11)  85 (15) | 703 (76)  97 (10)  126 (14) | 0.2383 |
| **Socioeconomic status**  1 (lowest)  2  3  4  5 (highest) | 116 (22)  147 (28)  85 (16)  93 (18)  83 (16) | 123 (22)  104 (19)  108 (19)  114 (21)  106 (19) | 131 (14)  178 (19)  162 (17)  220 (24)  235 (25) | <0.0001 |
| **Histology**  Ductal  Lobular  Other/Unknown | 401 (77)  48 (9)  75 (14) | 417 (75)  58 (10)  80 (14) | 688 (74)  104 (11)  134 (14) | 0.8092 |
| **Differentiation**  Low  Intermediate  High  Unknown | 145 (28)  257 (49)  96 (18)  26 (5) | 190 (34)  236 (43)  104 (19)  25 (4) | 316 (34)  417 (45)  149 (16)  44 (5) | 0.1492 |
| **Comorbidity burden (Charlson Comorbidity Index, excluding breast cancer in the count)**  0  1  ≥2 | 410 (78)  56 (11)  58 (11) | 519 (93)  20 (4)  16 (3) | 899 (97)  15 (2)  12 (1) | <0.0001 |
